# Ligase Pellino3 Regulates Macrophage Action and Survival in Response to VSV Infection in RIG-I-Dependent Path

**DOI:** 10.1155/2021/6668463

**Published:** 2021-07-01

**Authors:** Patryk Reniewicz, Anna Kula, Edyta Makuch, Michał Ochnik, Tomasz Lipiński, Jakub Siednienko

**Affiliations:** ^1^Bioengineering Research Group, Łukasiewicz Research Network–PORT Polish Center for Technology Development, Wroclaw 54-066, Poland; ^2^Laboratory of Medical Microbiology, Ludwik Hirszfeld Institute of Immunology and Experimental Therapy, Polish Academy of Sciences, Wroclaw 53-114, Poland; ^3^Laboratory of Virology, Ludwik Hirszfeld Institute of Immunology and Experimental Therapy, Polish Academy of Sciences, Wroclaw 53-114, Poland

## Abstract

Sensing of viral particles and elements that initiate mechanisms of immune response is an intrinsic ability of mammalian cells. Regulatory cytokines and antiviral mediators are released after triggering of complex signaling cascades in response to interaction of pathogen particles with pattern recognition receptors (PRRs) leading to the production of interferons (IFN) and proinflammatory cytokines. Viral RNA in the cytoplasm constitute a potent danger molecule that recognition is performed by RIG-I-like receptors, the most common group of receptors in mammalian cells, capable to recognize a foreign RNA. It is known that the E3 ubiquitin ligase Pellino3 plays an important role in antibacterial and antiviral response, but its involvement in the RLR pathways remains poorly understood. In this study, we investigate the molecular mechanisms of the innate immune response in BMDMs (immortalized macrophages from mouse bone marrow) during VSV infection. Here, we present evidence that the activation of the RIG-I/Pellino3/ERK1/2 pathway in BMDMs is crucial for the protection against VSV. We demonstrate that during infection, viral particles replicate in Pellino3 knockout BMDMs more effectively than in wild-type cells. Increased viral replication resulting in cell lysis and death is aid by impaired synthesis of IFN-I and inflammatory cytokines as a consequence of disturbances in the ERK1/2 pathway regulation.

## 1. Introduction

Cell growth inhibition and death is a common outcome of virus infection. In certain cases, cell death curbs virus replication. In others, cell death enhances virus dissemination and contributes to tissue injury, exacerbating viral disease. To control an ongoing infection, immune system has developed antiviral sensors generally known as pattern recognition receptors (PRRs) including Toll-like receptors (TLRs), RNA-dependent protein kinase (PKR), and cytosolic receptors. Molecular interactions between PRRs and microbial and viral particles result in the initiation of signaling cascade regulating the production and secretion of several cytokines including interferons (IFNs), tumor necrosis factor (TNF), and immunoregulatory chemokines [1].

One of the common pathogen-associated molecules that can be found in the cytoplasm of the infected cell is viral RNA. It can either arise from of viral genome upon its entry into a cell or can be de novo synthesized during the replication process. This foreign RNA is then sensed by specialized receptors; among them, the most important belongs to the family of RIG-I-like receptors (RLRs) [2–4]. RLRs are able to sense RNA from number of viruses, such as measles, Newcastle disease virus, influenza virus A, Ebola, Epstein-Barr virus, encephalomyocarditis virus (EMCV), vaccinia virus, and vesicular stomatitis virus (VSV) [5–13]. The signal from RLRs propagates downstream through the adapter protein named mitochondrial antiviral signaling protein (MAVS) [14, 15].

So far, two RIG-I/MAVS downstream major pathways of signal transduction have been identified. First pathway leads to the activation of TRAF3 (TNF receptor-associated factor), TANK (TRAF family member-associated NF-*κ*B activator), and TBK1 (TANK-binding kinase) proteins followed by the translocation of transcription factors IRF3 and/or IRF7 (interferon regulatory factor) to the nucleus. This directly leads to the induction of type I interferons (IFN-I) [15, 16]. Second pathway results in NF-*κ*B (nuclear factor *κ*-light-chain-enhancer of activated B cells) regulated production of proinflammatory cytokines. In this case, signal from adapter protein is transmitted to TRAF2 and TAK1 (TGF*β*-activated kinase) [17, 18]. Postulated application of vaccinia virus, Newcastle disease virus, or VSV in oncolytic therapy [19] prompts research on detailed understanding of the signaling pathways and processes induced by viruses.

Pellino3 is a ubiquitin ligase that regulates an activation process of MAP (mitogen-activated protein) kinases, like ERK1/2 (extracellular signal-regulated kinases 1/2), p38, and JNK (c-Jun *N*-terminal kinases) [20–22]. In addition, it has been proven that Pellino3 is a regulator of the signaling pathways of the various receptors. In the TNFR1 (tumor necrosis factor receptor) signaling pathway, Pellino3 can target RIP1 (receptor-interacting serine/threonine protein kinase) after TNF treatment and thus prevents DISC (death-inducing signaling complex) formation and cell apoptosis [23]. It also has been shown that Pellino3 ubiquitinates RIP2 and activates the molecule, stimulating further signal propagation. Consequently, Pellino3 induces NOD2-dependent (nucleotide-binding oligomerization domain containing) production of cytokines, chemokines, and bactericidal peptides [24]. Moreover, it can affect the TLR3 pathway after EMCV infection by ubiquitination of TRAF6 that blocks the signal transmission to IRF7, resulting in shutdown of IFN-I secretion [25]. Furthermore, there are some evidence that Pellino3 is involved in negative regulation of the TLR2 and TLR4 signaling after stimulation with LPS (lipopolysaccharides) [26, 27].

Here, we show for the first time that in immortalized BMDM cell line, Pellino3 is an important element of RLR-dependent production of the IFN-I and other cytokines after VSV infection and this process is regulated by phosphorylation of ERK1/2 kinases. Moreover, we show that inhibition of ERK1/2 following VSV infection results in the suppression of the cytokine production and increased macrophage mortality. We also found that decreased cytokine production and cell viability of *Peli3^−/−^* BMDMs correlate with the higher virus titer observed in infected Pellino3 knockout macrophage cell cultures.

## 2. Materials and Methods

### 2.1. Cell Culture, Viruses, and Reagents

Immortalized BMDM cell lines from wild-type (WT), *Peli3^−/−^*, and *MAVS^−/−^* mice were gifts from Professor Paul N. Moynagh (National University of Ireland, Maynooth, Ireland). B16-Blue IFN-*α*/*β*, A549-Dual, A549-Dual KO-MDA-5, and A549-Dual KO-RIG-I cells were purchased from Invivogen (San Diego, CA, USA). Cells were grown in DMEM with GlutaMAX (Gibco, Gaithersburg, MD, USA) (BMDMs and all A549 cell lines) and in RPMI with GlutaMAX (B16s) supplemented with 10% heat-inactivated foetal bovine serum (Sigma, St. Louis, MO, USA) and Normocin (Invivogen, San Diego, CA, USA) and maintained at 37°C in a humidified atmosphere of 5% CO_2_. In addition, B16-Blue IFN-*α*/*β* cells required a selective antibiotic, Zeocin (100 *μ*g/ml) (Invivogen, San Diego, CA, USA); A549-Dual cell lines required Zeocin (100 *μ*g/ml) and blasticidin (10 *μ*g/ml) (Invivogen, San Diego, CA, USA). A wild-type Indiana VSV (vesicular stomatitis virus) serotype was originally obtained from Dr. C. Buckler (National Institutes of Health, Bethesda, MD, USA). Antibodies anti-p38 MAPK, anti-phospho-p38 MAPK (Thr180/Tyr182), anti-p44/p42 MAPK ERK1/2, anti-phospho-p44/p42 MAPK ERK1/2 (Thr202/Tyr204), anti-SAPK/JNK, and anti-phospho-SAPK/JNK (Thr183/Tyr185) were purchased from Cell Signaling (Danvers, MA, USA); secondary antibody IRDye 800CW Goat anti-Rabbit IgG (H+L) was purchased from LI-COR (Lincoln, NE, USA). Inhibitor FR180204 was purchased from Sigma (St. Louis, MO, USA).

### 2.2. Cell Treatments

Before experiment, cells were trypsinized (Sigma, St. Louis, MO, USA) and counted in TC20 automated cell counter (Bio-Rad, Hercules, CA, USA) with trypan blue (Sigma, St. Louis, MO, USA). BMDMs were seeded in various densities dependent on the following tests (1 × 10^6^ cells/ml for ELISA, type I IFN bioassay; 5 × 10^5^ cells/ml for cell viability assay; and 1.5 × 10^6^ cells/ml for Western blotting), and next, cells were cultured for 24 hours at 37°C in a humidified atmosphere of 5% CO_2_. Then, cultures were infected with VSV at MOI 0.015 and/or 1.5 and incubated for various time periods, dependent on following experiment: 48 h for cell viability assay; overnight for ELISA and type I IFN bioassay or for 0.5, 1, 2, 4, and 6 hours to Western blotting. If experiment required inhibitor (FR180204) administration, it was added one hour before viral infection at concentration 2.0 *μ*M (for ELISA and type I IFN bioassay) or 5 *μ*M (for cell viability assay). Additionally, inhibitor was added 24 h of postinfection for cell viability assay. Mock cells were pretreated with DMSO. A549-Dual, A549-Dual KO-MDA-5, and A549-Dual KO-RIG-I cells were seeded at a density of 2.8 × 10^5^ cells/ml for IRF pathway reporter assay. B16-Blue IFN-*α*/*β* was seeded at a density of 4.2 × 10^5^ cells/ml for type I IFN bioassay. Cells were cultured for 24 hours at 37°C in a humidified atmosphere of 5% CO_2_ before appropriate assay.

### 2.3. Sample Preparation

In ELISA and bioassay tests, medium above infected cells were collected and centrifuged at 300*g* for 5 minutes to remove cells debris. If necessary, samples were frozen at -80°C. In Western blotting, medium above infected cells were aspirated and discarded. Cells were rinsed two times with PBS (Sigma, St. Louis, MO, USA) and lysed in 75 *μ*l HS buffer (50 mM HEPES pH 7.5, 150 mM NaCl, 2 mM EDTA pH 8, 1% NP-40, 0.5% sodium deoxycholate) supplemented with proteinase inhibitors cOmplete Mini Tablets (Roche, Basel, Switzerland) and phosphatase inhibitors PhosSTOP (Roche, Basel, Switzerland) and incubated for 20 minutes on ice. Next, to cell lysates, 75 *μ*l twice concentrate Laemmli buffer (Bio-Rad, Hercules, CA, USA) and 7.5 *μ*l 2-mercaptoethanol (Sigma, St. Louis, MO, USA) were added and incubated at 99°C for 10 minutes. If necessary, samples were frozen at -20°C.

### 2.4. Total RNA Isolation

Cells after viral infection were lysed in 0.5 ml of PureZOL (Bio-Rad, Hercules, CA, USA) directly in a culture dish. Next, cell lysates were either frozen in -20°C or immediately processed according to the manufacturer's instructions of PureZOL.

### 2.5. First-Strand cDNA Synthesis

Isolated RNA (1 *μ*g) was incubated with DNase I (Thermo Fisher Scientific, Waltham, MA, USA) at 37°C for 30 min. Then, DNase I was double inactivated by addition of EDTA and incubation at 60°C for 10 min. Thereafter, cDNA was synthesized using iScript reverse transcription supermix for RT-PCR (Bio-Rad, Hercules, CA, USA), accordingly with the manufacturer's instructions. Reactions were incubated at 25°C for 5 min followed by 37°C for 40 min followed by 46°C for 40 min and heated to 95°C for 1 min. Obtained cDNA was either frozen at -20°C or immediately used for PCR reactions.

### 2.6. PCR

Total cDNA (10 ng) was used as a starting material for qPCR with CFX Connect qPCR system (Bio-Rad) and iTaq Universal SYBR Green Supermix (Bio-Rad). For the amplification of the *Peli3* gene, the following primers were used: forward: CAAGGGCTCTTGCGTTCTCT and reverse: CCCAGGACGATGAGTTCACC. For each mRNA quantification, the housekeeping gene hypoxanthine phosphoribosyltransferase 1 (HPRT) was applied as a reference point using the following primers: *Hprt*, forward: AGGGATTTGAATCACGTTTG and reverse: TTTACTGGCAACATCAACAG. Real-time PCR data were analyzed using the 2^−ΔΔCT^ method. Conventional PCR was performed using DNA REDTaq polymerase (Sigma, St. Louis, MO, USA) with 70 ng of total cDNA according to the manufacturer's protocol. For the amplification of the specific genes, the following primers were used: *Ddx58*, forward: ACAAACCGGGCAACAGGAAT and reverse: CAATGCCTTCATCAGCGACC; *Ifih1*, forward: AACACGACAGAGCACCTACG and reverse: TTGTTCAGTCTGAGTCATGGGC; and *Mavs*, forward: CGCAGCAAATGTTGCCTCTG and reverse: TTTGTCCTCAGGGCAGTACG. PCR products were resolved by 1.5% (*w*/*v*) agarose gel electrophoresis and then analyzed using a Gel Doc (Bio-Rad, Hercules, CA, USA).

### 2.7. Western Blotting

Cell lysates (described above) were subjected to SDS-PAGE in 12% Mini-PROTEAN TGX Stain-Free Gel (Bio-Rad, Hercules, CA, USA), at low voltage of 100 V for 85-95 minutes in Tris/glycine buffer (Bio-Rad, Hercules, CA, USA) witch addition of 0.1% SDS. Next, proteins separated in a gel were transferred to nitrocellulose membranes, 0.45 *μ*m (Bio-Rad) in Trans-Blot Turbo transfer system (Bio-Rad, Hercules, CA, USA) at 1.0 A and 25 for 30 minutes. Membranes were blocked in casein blocking buffer (Sigma, St. Louis, MO, USA) for 1 hour at room temperature. Next, membranes were probed with indicated primary antibodies overnight at 4°C. After three washes with TBS-T (50 mM Tris, 150 mM NaCl, and 0.05% Tween 20), membrane was incubated with corresponding secondary antibody for 1 hour at room temperature. After another three washes with TBS-T, the membrane was scanned by Odyssey CLx Infrared Imaging System (LI-COR, Lincoln, NE, USA).

### 2.8. Type I IFN Bioassay

WT and *Peli3^−/−^* BMDMs were seeded in 96-well plates and stimulated as indicated. Detection of VSV-induced bioactive murine type I IFN was assessed using B16-Blue IFN-*α*/*β* cells, essentially as described by the manufacturer (Invivogen, San Diego, CA, USA).

### 2.9. ELISA

The protein concentrations of TNF, RANTES, and IP-10 in a postinfectious supernatant from WT and *Peli3^−/−^* BMDMs were measured by DuoSet ELISA according to the manufacturer's instruction (R&D System, Minneapolis, MN, USA). Samples for test were diluted two times. All ELISA tests were performed by automated system E-LizaMat X-2 (DRG International, Springfield, NJ, USA).

### 2.10. VSV Replication Assessment

WT and *Peli3^−/−^* BMDMs were seeded in 6-well plates. After 24 h, cells were infected with VSV at MOI 0.5. 24 h of postinfection, cell culture supernatant was collected and for the determination of virus titer conducted according to TCID50 method on L929 culture [28].

### 2.11. alamarBlue Cell Viability Assay

WT and *Peli3^−/−^* BMDMs were seeded in 96-well plates. After 24 h, cells were infected with VSV at MOI 0.015 and 1.5. 48 h or 72 h of postinfection, cell supernatants were removed, and the cells were incubated with 10% alamarBlue for 4 h at 37°C followed by fluorescence measurement, at excitation 560 nm and emission 590 nm, with Synergy H4 Hybrid Microplate Reader (BioTek, Winooski, VT, USA).

### 2.12. IRF Pathway Reporter Assay

A549-Dual, A549-Dual KO-MDA-5, and A549-Dual KO-RIG-I were seeded in 96-well plates and infected after 24 h with VSV at MOI 1.5. IRF pathway activation was determined after 16 h following the manufacturer's instruction (Invivogen, San Diego, CA, USA).

### 2.13. Data Analysis

Statistical analysis was carried out using the unpaired Student *t*-test using GraphPad Prism 7.04. *p* values of less than or equal to 0.05 were considered to indicate a statistically significant difference (^∗^*p* ≤ 0.05, ^∗∗^*p* ≤ 0.01, and ^∗∗∗^*p* ≤ 0.001).

## 3. Results

### 3.1. Antiviral Response in VSV-Infected BMDMs Prevents Pathogen-Induced Cell Lysis in Pellino3/RIG-I-Dependent Manner

To investigate the molecular mechanism by which the immune system detects neurotropic viruses, we used VSV, an arthropod-borne rhabdovirus that causes fatal paralytic disease in mammals, including mice [29]. Previous work has shown that following peripheral inoculation in mice, VSV is captured by lymph nodes macrophages, preventing systemic infection/hematogenous dissemination [30] so we reasoned to carry out the research on immortalized BMDM cell line.

**Figure 1:**
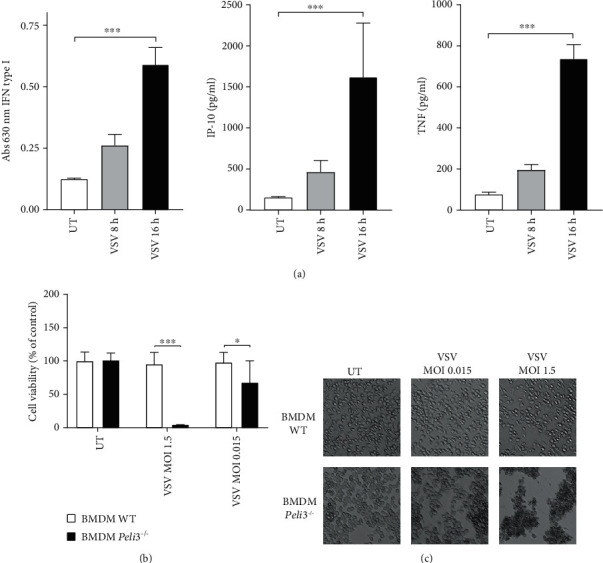
Lack of Pellino3 results in VSV-induced disintegration of BMDMs. (a) WT BMDMs were infected with VSV at MOI 1.5. Cell supernatants were collected 8 and 16 h of postinfection. Type I IFN was measured by bioassay; level of IP-10 and TNF was measured by ELISA as described under Materials and Methods. (b, c) WT and *Peli3^−/−^* BMDMs were infected with VSV at MOI 1.5 and 0.015. Cell viability was evaluated by alamarBlue test 48 h of postinfection. Photographs were taken using 10x microscope objective. Results are representative of at least three independent experiments performed in triplicate (mean ± S.E.). Statistical analysis was carried out using the unpaired Student *t*-test using GraphPad Prism 7.04. *p* values of less than or equal to 0.05 were considered to indicate a statistically significant difference (^∗^*p* ≤ 0.05, ^∗∗^*p* ≤ 0.01, and ^∗∗∗^*p* ≤ 0.001).

We measured VSV-induced cytokine production by type I IFN bioassay or ELISA, and we demonstrated that treatment of WT BMDMs with VSV resulted in accumulation of IFN-I, IP-10, and TNF in a time-dependent manner (Figure 1(a)).

Next, taking into account the cytolytic properties of this virus, we looked at macrophages' resistance to viral lysis. WT and *Peli3^−/−^* BMDMs were infected with different MOI of VSV and incubated for 48 hours, and cell viability was evaluated by alamarBlue test. The significant difference in the cell viability was observed indicating that VSV is more virulent to *Peli3^−/−^* BMDMs compared with WT (Figure 1(b)). Inverted microscope photos (Figures 1(c)) correlate with the results of the alamarBlue test and show strong morphological changes in *Peli3^−/−^* BMDMs after VSV infection.

**Figure 2:**
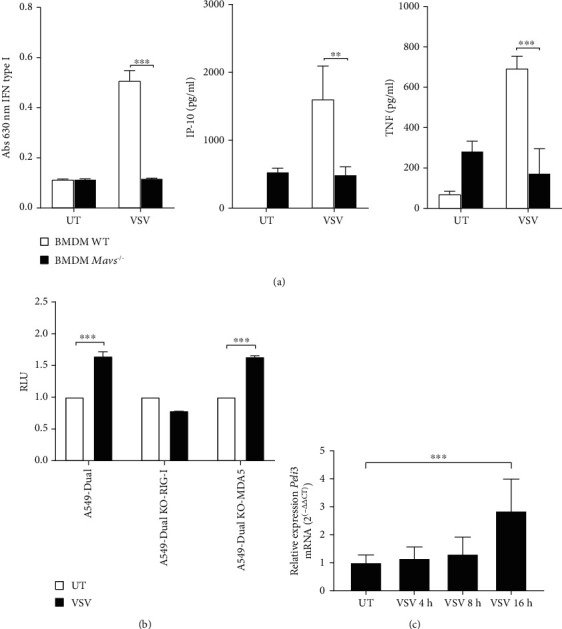
Immune response in BMDMs after VSV infection is RIG-I-dependent. (a) Wild-type and *Mavs^−/−^* BMDM cells were infected with VSV at MOI 1.5. Cell culture supernatants were collected 16 h of postinfection. Type I IFN concentration was measured by bioassay; level of IP-10 and TNF was measured by ELISA as described under Materials and Methods. (b) A549-Dual, A549-Dual KO-MDA-5, and A549 KO-RIG-I cells were infected with VSV at MOI 1.5. After 16 h of the interferon regulatory factor (IRF) pathway was determined by monitoring activity of Lucia luciferase as described under Materials and Methods. Results are representative of at least three independent experiments performed in triplicate (mean ± S.E.). (c)WT BMDMs were infected with VSV at MOI 1.5 and incubated for 4, 8, and 16 h. Therefore, total RNA was isolated, converted to first-strand cDNA, and used as a template for quantitative real-time PCR as described under Materials and Methods. Quantitative real-time PCR was used to assay the expression levels of *Peli3*. Experiments were repeated at least three times, and data are presented in relative expression units where *Hprt* was used to normalize all samples and untreated cells were assigned an arbitrary value of 1. Statistical analysis was carried out using the unpaired Student *t*-test using GraphPad Prism 7.04. *p* values of less than or equal to 0.05 were considered to indicate a statistically significant difference (^∗^*p* ≤ 0.05, ^∗∗^*p* ≤ 0.01, and ^∗∗∗^*p* ≤ 0.001).

In order to determine whether VSV-induced production of IFN-I, IP-10, and TNF is dependent on RIG-I-like receptors (RLRs), we compared the response in WT and *Mavs^−/−^* BMDMs, taking into account that Mavs (mitochondrial antiviral signaling protein) is the key adapter of RLR receptors. To solve this, WT and *Mavs^−/−^* BMDMs were infected with VSV virus at MOI 1.5 and cytokines were measured by bioassay and ELISA. Results demonstrate that whereas stimulation of WT BMDMs with the VSV effected in IFN-I, IP-10, and TNF activation, a significantly lower induction of those cytokines was evident in *Mavs^−/−^* BMDMs (Figure 2(a)). These findings suggest that RLRs are the main group of receptors involved in the recognition of VSV in BMDM cells.

Next, we attempted to evaluate which RLR receptors are responsible for VSV virus recognition. For this purpose, we tested response to VSV infection in A549-Dual, A549-Dual KO-RIG-I, and A549-Dual KO-MDA5 cells that express the luciferase gene under the control of promoters activated by transcription factors from the IRF family. Luciferase production level after pathogen recognition is correlated with the level of antiviral response induced by receptors from the RLR family. We have shown that in both cell lines (A549-Dual and A549-Dual KO-MDA5), the IRF family transcription factors are activated in response to infection with VSV. However, we did not observe similar effect in A549-Dual KO-RIG-I cells, which confirms the involvement of the RIG-I receptor in the VSV-activated antiviral response (Figure 2(b)).

In parallel, we showed that the level of *Peli3* mRNA increased during VSV infection (Figure 2(c)) and corresponded with elevated secretion of IFN-I, IP-10, and TNF from WT BMDMs infected with VSV.

### 3.2. Absence of Pellino3 Ligase Results in a Lower Level of Cytokine Secretion and Increased Virus Multiplication in BMDMs

**Figure 3:**
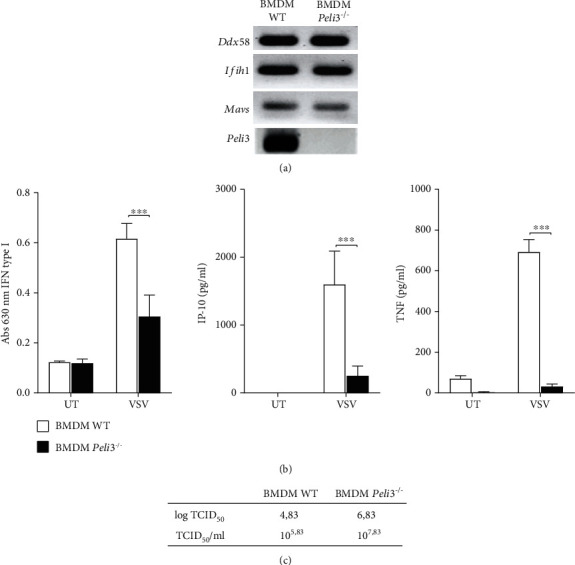
Pellino3 knockout causes decreased cytokine secretion and increased virus multiplication. (a) Total RNA was isolated from WT and *Peli3^−/−^* BMDMs and converted to first-strand cDNA. This was used as a template for conventional PCR amplifying genes as indicated. Products were resolved as described under Materials and Methods. (b) Wild-type and *Peli3^−/−^* BMDMs were infected with VSV at MOI 1.5. Cell culture supernatants were collected 16 h of postinfection. Concentration of type I IFN was measured by bioassay; level of IP-10 and TNF was measured by ELISA as described under Materials and Methods. (c) Wild-type and *Peli3^−/−^* BMDM cells were infected with VSV at MOI 0.5. Cell supernatants were collected 24 h of postinfection to determine viral titer as described under Materials and Methods. Results are representative of at least three independent experiments performed in triplicate (mean ± S.E.). Statistical analysis was carried out using the unpaired Student *t*-test using GraphPad Prism 7.04. *p* values of less than or equal to 0.05 were considered to indicate a statistically significant difference (^∗^*p* ≤ 0.05, ^∗∗^*p* ≤ 0.01, and ^∗∗∗^*p* ≤ 0.001).

Considering the results described above and fact that Pellino3 may affect antiviral signaling pathways [24], we aimed to investigate the effect of this ligase on RIG-I-dependent IFN-I activation and cytokine production. Bioassay and ELISA results showed that the productions of biologically active type I interferon and other tested cytokines after infection with VSV are reduced in *Peli3^−/−^* BMDMs (Figure 3(b)). Furthermore, we analyzed expression level of gene coding members of RLR family and their adapter protein. In both WT and *Peli3^−/−^* BMDMs, *Ddx58*, *Ifih1*, and *Mavs* mRNA levels were similar, indicating that the reported differences in cytokine induction are not associated directly with gene expression levels (Figure 3(a)) but rather are regulated by signal transduction. Moreover, we observed that decreased cytokine production correlates with increased viral replication in *Peli3^−/−^* BMDMs (Figure 3(c)).

### 3.3. Pellino3 Regulates Activation of ERK1/2 in BMDMs after VSV Infection

**Figure 4:**
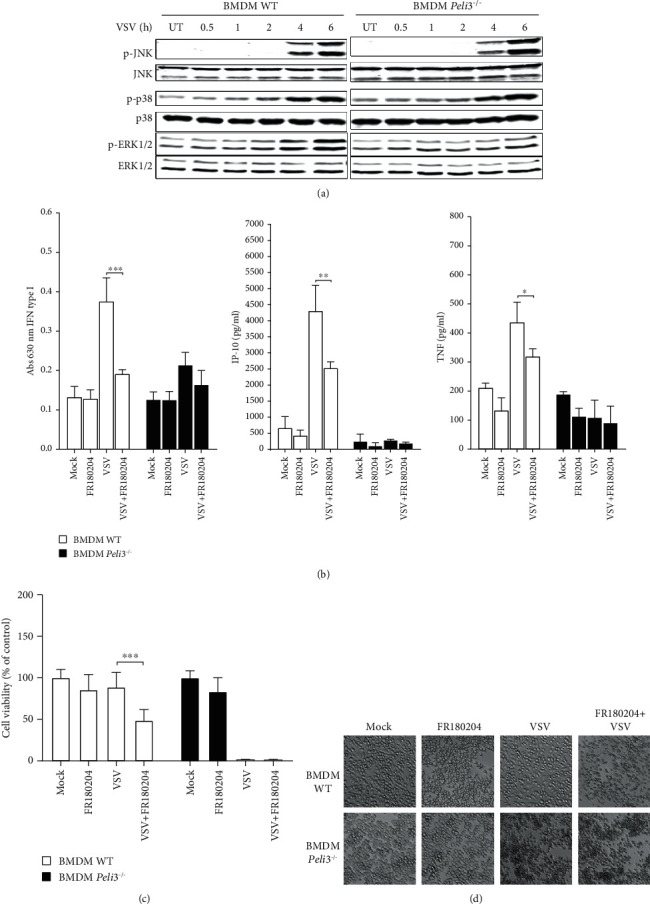
VSV-dependent ERK1/2 activation and VSV resistance are regulated by Pellino3. Wild-type and *Peli3^−/−^* BMDMs were pretreated with DMSO (Mock) or FR180204 (2.0 *μ*M for 1 h (b) or 5 *μ*M for 72 h (c, d)). Next, the cells were infected with VSV at MOI 1.5 (a, b, c, d). (a) Cell lysates were subjected to SDS-PAGE followed by Western blotting. Protein detection was performed using specific antibodies and appropriate secondary antibodies conjugated to the fluorescent dye in the infrared range. Visualization was performed using the Odyssey CLx Imaging System LI-COR. The results presented are representative of at least three independent experiments. (b) Cell supernatants were collected 16 h of postinfection. Type I IFN was measured by bioassay; level of IP-10 and TNF was measured by ELISA as described under Materials and Methods. (c) Cell viability was evaluated by alamarBlue test 72 h of postinfection. (d) Photographs were taken using 10x microscope objective. Results are representative of at least three independent experiments performed in triplicate (mean ± S.E.). Statistical analysis was carried out using the unpaired Student *t*-test using GraphPad Prism 7.04. *p* values of less than or equal to 0.05 were considered to indicate a statistically significant difference (^∗^*p* ≤ 0.05, ^∗∗^*p* ≤ 0.01, and ^∗∗∗^*p* ≤ 0.001).

Described signaling pathways in which Pellino3 activity was confirmed are related with MAP kinase paths [21]. Therefore, we reasoned to examine phosphorylation pattern of MAP kinases p38, JNK, and ERK1/2. Whole cell lysates from VSV-infected BMDMs were prepared, and the phosphorylation patterns of selected MAP kinases were analyzed by Western blotting. Interestingly, we found that neither JNK nor p38 phosphorylation was altered and only phosphorylation of ERK1/2 in Pellino3-deficient cells was suppressed (Figure 4(a)).

To assess the potential of ERK1/2 to mediate RIG-I-dependent gene activation, we examined the secretion of IFN-I, IP-10, and TNF in WT and *Peli3^−/−^* BMDMs infected with VSV in the presence or absence of a specific inhibitor of these kinases—FR180204 [31]. We found that the production of type I interferon and proinflammatory cytokines by WT BMDMs is decreased after administration of FR180204 comparably in Pellino3-deficient cells and WT cells (Figure 4(b)). This result is consistent with our findings from Western blot analysis and suggests that phosphorylation of ERK1/2 is a key point in the regulation of the VSV-induced RIG-I-dependent signaling pathway by Pellino3. Taken together, these results suggest that Pellino3 acts as a positive regulator in activation of the ERK1/2 pathway.

Additionally, to ensure that the ERK1/2 and Pellino3-dependent cytokine expression aids resistance to VSV, we examined the viability of BMDM cells pretreated with the inhibitor FR180204 and then infected with VSV. As shown in Figure 4(c), decreased cell viability of WT BMDMs was observed in response to VSV infection after treatment with the inhibitor. The result was further confirmed by examination of images from an inverted microscope (Figure 4(d)) where signs of morphological changes are evident in WT BMDMs pretreated with ERK1/2 inhibitor. High mortality of VSV-infected *Peli3^−/−^* BMDMs was independent of inhibitor treatment according to alamarBlue cell viability assay and microscopic images.

Taken together, these findings clearly indicate that the antiviral mechanism of ERK1/2 activation in macrophages is triggered by VSV/RIG-I and regulated by Pellino3 ligase.

## 4. Discussion

Over the past decade, intracellular RLR family receptors that recognize exogenous RNA have gained increasing interest from scientists. The available literature data indicate RLR contribution in the activation of interferon type I production and cytokines such as TNF and IP-10 [15–18]. However, signaling cascades triggered after virus recognition by RLR receptors are not fully characterized and remain the area of interest to many research groups. In this study we indicate that Pellino3 ligase is capable of modifying the RLR-dependent signaling pathways after VSV infection. We show for the first time that Pellino3 knockout macrophages are unable to activate an immune response after viral infection and to prevent virus-induced cell lysis.

It has been shown that VSV infection results in elevated production of proinflammatory cytokines [32]. Our research showed that BMDMs infected with VSV produce proinflammatory cytokines such as TNF and IP-10, as well as biologically active interferon type I and that the cytokine concentration depends on the stimulation time. We have observed up to four times higher level of cytokines produced during 16 hours of infection compared to the level observed after 8 hours of postinfection (Figure 1(a)).

It is known that the detection of RNA virus by RLRs results in recruitment of adapter protein MAVS followed by activation of signaling cascades leading to the induction of antiviral and inflammatory mediators [14, 15, 33]. Thus, to determinate the involvement of cytosolic receptors in VSV recognition, *Mavs^−/−^* BMDMs have been studied as a model. Using type I IFN bioassay and ELISA methods, the level of IFN-I, IP-10, and TNF in BMDMs after VSV infection has been confirmed to relay on the presence of MAVS adapter (Figure 2(a)). Therefore, obtained data clearly demonstrate that in BMDMs, activation of immune response after VSV infection depends on the recognition of viral RNA by RLRs.

Given that MAVS activation requires the signal from cytosolic receptors (RIG-I or MDA-5) [34], we decided to examine which one of them is involved in VSV recognition. For this purpose, we applied human A549-Dual RIG-I KO and MDA5 KO reporter cell lines. The cell lines were modified to stably express a synthetic gene that encodes a luciferase gene controlled by the minimal isg54 promoter (interferon-stimulated gene 54) and five ISRE sequences (interferon-sensitive response element). This system enables facile monitoring of the transcription factor activation from the IRF family, essential during viral infection [35]. We observed the lack of activation of luciferase expression in cells A549-Dual RIG-I KO after a viral infection, while in cells A549-Dual MDA5 KO after VSV infection, activation of luciferase expression was at the same level as in the control cell line (Figure 2(b)). Thus, we concluded that in A549 cells, after VSV infection, IRF transcription factors are activated in RIG-I receptor-dependent path. In addition, it has been shown that in response to viral infection, the expression of Pellino3 encoding gene is increased (Figure 2(c)). This observation corresponds well with published results, which show that the Pellino3 protein is expressed at a higher level after Toll-like receptor 3 stimulation, which recognizes viral dsRNA [25].

Next, the role of Pellino3 in IFN-I and cytokine regulation during VSV infection was studied. It was shown that RIG-I-induced IFN-I, IP-10, and TNF secretion requires Pellino3 and that this process is abolished in *Peli3*^−/−^ BMDMs (Figure 3(b)). The expression level of *Ddx58*, *Ifih1*, and *Mavs* genes was analyzed to confirm that reported differences in cytokine induction are not related to expression of RLRs and their adapter protein but are caused by disturbances in signal transduction (Figure 3(a)). Moreover, we found that failure in stimulating macrophages to produce antiviral agents results in an increased amount of replicated VSV in *Peli3*^−/−^ BMDMs (Figure 3(c)). It was also observed that VSV infection of *Peli3^−/−^* BMDMs led to the death of these cells, while VSV-infected WT BMDMs were not lysed (Figures 1(b) and 1(c)).

To address these unintuitive differences in antiviral action of macrophages, phosphorylation pattern of MAP kinases was examined (Figure 4(a)). It has been already published that in some cells, JNK and p38 MAP kinases are activated in response to VSV infection [20–22], which is in line with our results obtained from VSV-infected BMDMs. Additionally, we have shown for the first time that Pellino3 is necessary to activate phosphorylation of ERK1/2 kinases after VSV infection. Next, necessity of ERK1/2 phosphorylation for proper production of IFN-I and cytokines was confirmed. The treatment with FR180204, a specific ERK1/2 inhibitor, caused a decreased proinflammatory response during VSV infection in BMDMs (Figure 4(b)). Those results strongly indicate that Pellino3 is a key regulator of ERK1/2 phosphorylation which facilitates ability of BMDMs to produce type I interferon and proinflammatory cytokines. Finally, it was confirmed that absence of Pellino3 and pharmacological suppression of the ERK1/2 pathway result in suppression of BMDM antiviral response that leads to increased amount of VSV multiplication and cell lysis (Figures 4(c) and 4(d)).

VSV, as one of the oncolytic viruses, has the ability to kill cancer cells. Oncolytic action is based largely on impaired INF production in cancer cells, which is a key reason of greater susceptibility to VSV infection [19, 32, 36]. Indication and characterization of the pivotal proteins involved in VSV-induced response is essential element of optimizing anticancer therapy. Our study showing that VSV infection leads to the RIG-I/Pellino3/ERK1/2-dependent secretion of IFN-I and proinflammatory cytokines contributes to a better understanding of the immunoregulation of antiviral response during VSV infection. The results may positively contribute to the improvement of safety and efficacy of VSV-based anticancer therapy.

## 5. Conclusions

In this study, we indicate that Pellino3 plays an important role in RIG-I-dependent production of the IFN-I and proinflammatory cytokines in VSV-infected macrophages. Our results show that VSV stimulation leads to upregulation of Pellino3 expression. Simultaneously, we observed Pellino3-dependent activation of ERK1/2, which is essential for IFN-I, IP-10, and TNF synthesis.

Based on this, we suggest that after recognition of VSV by RIG-I, the increasing expression of Pellino3 and activation of ERK1/2 in macrophages can drive IFN-I and cytokine secretion aiding protection against VSV infection, while the depletion of Pellino3 or inhibition of ERK1/2 activation results in increased viral multiplication and cell death.

## Data Availability

The data that support the findings of this study are available from the corresponding author upon reasonable request.

## Abbreviations

BMDMs: Immortalized bone marrow-derived macrophages from primary murine bone marrow cells
ERK 1/2: Extracellular signal-regulated kinases 1/2
IFN-I: Type I interferons
MAVS: Mitochondrial antiviral signaling protein
MAP: Mitogen-activated protein
RLR: RIG-I-like receptor
TLR: Toll-like receptor
TNF: Tumor necrosis factor
IP-10: Interferon gamma-induced protein 10 or C-X-C motif chemokine ligand 10 (CXCL10).
